# Ratio of C-Reactive Protein to Albumin Predicts Muscle Mass in Adult Patients Undergoing Hemodialysis

**DOI:** 10.1371/journal.pone.0165403

**Published:** 2016-10-21

**Authors:** Te-Chih Wong, Hsiu-Yueh Su, Yu-Tong Chen, Pei-Yu Wu, Hsi-Hsien Chen, Tso-Hsiao Chen, Yung-Ho Hsu, Shwu-Huey Yang

**Affiliations:** 1 School of Nutrition and Health Sciences, Taipei Medical University, Taipei, Taiwan, Republic of China; 2 Department of Dietetics, Taipei Medical University Hospital, Taipei, Taiwan, Republic of China; 3 Division of Nephrology, Department of Internal Medicine, Taipei Medical University Hospital, Taipei, Taiwan, Republic of China; 4 Division of Nephrology, Department of Internal Medicine, Wan Fang Hospital, Taipei Medical University, Taipei, Taiwan, Republic of China; 5 Division of Nephrology, Department of Internal Medicine, Shuang Ho Hospital, Taipei Medical University, Taipei, Taiwan, Republic of China; 6 Nutrition Research Center, Taipei Medical University Hospital, Taipei, Taiwan, Republic of China; Hospital Universitario de la Princesa, SPAIN

## Abstract

Recent studies have indicated that the ratio of C-reactive protein to albumin (CRP–Alb ratio) is associated with clinical outcomes in patients with disease. We examined the predictive value of this ratio in patients undergoing hemodialysis (HD). In this cross-sectional study, 91 eligible adult HD patients were analyzed, and the correlation between the CRP–Alb ratio and skeletal muscle mass normalized for body weight (SMM/wt; estimated using a bioelectrical impedance analyzer) was investigated. The mean age of the study participants was 54.9 ± 6.6 years (ranging from 27 to 64 years); 43 (47.2%) were men. The mean values for the SMM/wt were 39.1% ± 5.4%. The CRP–Alb ratio was found to be negatively correlated with SMM/wt (r = −0.33, *P* = 0.002) and creatinine (r = −0.20, *P* = 0.056). All the univariate significant and nonsignificant relevant covariates were selected for multivariable stepwise regression analysis. We determined that the homeostasis model assessment-estimated insulin resistance and CRP–Alb ratio were independent risk determinants for SMM/wt (β_HOMA-IR_ = −0.18 and β_CRP–Alb ratio_ = −3.84, adjusted R^2^ = 0.32). This study indicated that the CRP–Alb ratio may help clinicians in predicting muscle mass in adult patients undergoing HD.

## Introduction

Muscle wasting, which is frequently reported in patients with end-stage renal disease (ESRD), leads to morbidity and mortality risk [1–3]. There is a growing awareness that inflammation probably triggers muscle catabolism [4], which is accompanied by multifactorial complexities involving anorexia, energy expenditure, hormonal derangements, and metabolic alterations [5, 6]. Consequently, markers identified as a reflection of malnutrition, inflammation, or a combination of the two processes, are necessary.

In clinical practice, levels of serum albumin and high-sensitivity C-reactive protein (CRP) are prevalently monitored. Zimmerman et al. observed that albumin and CRP are independent predictors of all-cause mortality in patients undergoing hemodialysis (HD) [7]. CRP is an acute-phase protein synthesized by hepatocytes in response to stimuli from proinflammatory cytokines [8]. Higher CRP levels have been shown to be associated with lower serum albumin levels in patients with ESRD [9, 10]. Regarding muscle mass, Kaysen et al. determined that CRP levels are associated with reduced albumin and creatinine levels in dialysis patients with inflammation [11]. In addition, serum CRP levels are independently associated with sarcopenic obesity, which is characterized by low skeletal muscle mass (SMM) with high body fat in Korean women [12] and elderly Chinese men [13]. Furthermore, previous cross-sectional studies have shown a positive association between albumin and appendicular SMM in older adults [14]. Consequently, merged information regarding CRP and albumin, calculated as the ratio of CRP to albumin (CRP–Alb ratio), could serve as a predictive marker for evaluating clinical outcomes.

The CRP–Alb ratio, first proposed by Fairclough et al. [15], has been applied as a prognostic marker in patients with cancer [16–18] and as an independent risk factor for mortality in patients with sepsis [19]. However, this marker has rarely been employed in studies on ESRD patients. According to our research, no previous studies have investigated using the CRP–Alb ratio to predict muscle wasting in ESRD patients, which is prominent and associated with mortality. In this study, we performed cross-sectional analysis to investigate the predictive value of the CRP–Alb ratio and hypothesized that this ratio could be used as an independent risk determinant for muscle mass in HD patients.

## Methods

### Participants

**Fig 1:**
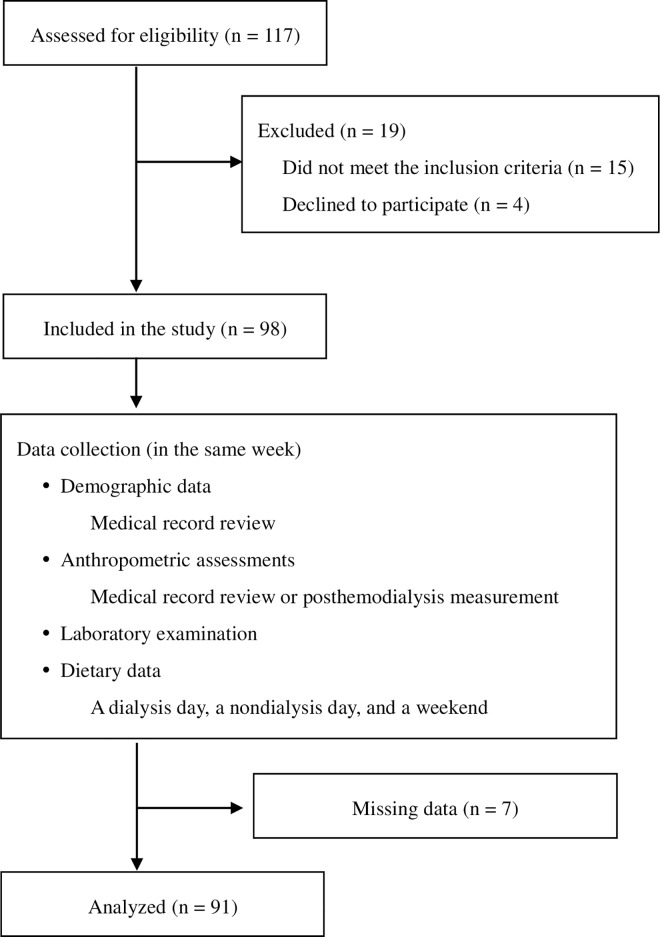
Flowchart indicating patient enrollment and study procedure.

This was a cross-sectional study; all data were collected in the same week by well-trained staff. Participants were recruited through advertisements in three hospital-based HD centers at Taipei Medical University (TMU) from September 2013 to January 2015. A total of 117 participants undergoing HD for at least 3 consecutive months were recruited. The criteria for inclusion were as follows: aged 20 to 64 years old; a minimum equilibrated Kt/V (eKt/V) of 1.2 for a thrice-weekly HD regimen; and absence of malignant tumors, cirrhosis, acute infection, and hospitalization 1 month prior to recruitment. Patients with severe edema, amputation, inadequate or excessive reported energy intake (< 500 kcal/day or > 3500 kcal/day), or missing assessment data were excluded. Participants were also required to have maintained their regular medication for at least 3 months prior to recruitment. All the standardized methods and procedures used in this study were specifically approved by the Research Ethics Committee of TMU (No. 201302024) and conducted according to the Declaration of Helsinki (S1 File). Written informed consent was obtained from all subjects before the commencement of the study. Ultimately, data for 91 consecutive adult patients were assessed (Fig 1).

### Demographic data and anthropometric assessment

By reviewing the patients’ medical charts, their age, sex, dialysis vintage, dialysis dose, and interdialytic weight gain, as well as their history of diabetes, hypertension, and cardiovascular disease were obtained for the demographic variables. In addition, height, and dry weight were determined for anthropometric data. We calculated the body mass index (BMI) as dry weight (in kilograms) adjusted for the square of height (in meters). After an HD session, SMM was determined as the sum of the total body muscle mass by using InBody S10 (Biospace, Seoul, Korea), a multifrequency bioelectrical impedance analyzer (BIA), and normalized for body weight (SMM/wt).

### Analyses of blood samples

Monthly routines and standard laboratory examinations were performed at the clinical laboratories of each hospital through automated and standardized methods. All preprandial and predialysis blood samples were collected after fasting for at least an 8-h period and subsequently analyzed. The preceding mean 3-month albumin (bromocresol green), random blood glucose, blood urea nitrogen, creatinine, total cholesterol, triglyceride, and intact parathyroid hormone levels were retrieved. The concentrations of high sensitivity CRP and insulin were determined at the clinical laboratory of TMU Hospital. In this study, the high sensitivity CRP were measured using a particle-enhanced immunoturbidimetric assay (IMMULITE®; Diagnostic Products Corp., Los Angeles, California), and the coefficient of variation was 5% at 0.02 mg/dL of CRP. The index of insulin resistance was indicated as the homeostasis model assessment-estimated insulin resistance (HOMA-IR) [20]. We also used the geriatric nutritional risk index (GNRI), which is a simplified, reliable and accurate index for identifying HD patients at nutritional risk [21], to evaluate the nutritional status of our participants. Previous studies have indicated that, regardless of sex and age, HD patients with albumin < 3.5 g/dL, high sensitivity CRP > 0.5 mg/dL, and GNRI < 91.2 received diagnoses of hypoalbuminemia, low-grade inflammation [22], and nutritional risk [21], respectively. In this study, the CRP–Alb ratio was defined as the serum CRP level divided by the serum albumin level.

### Estimation of dietary intake

Descriptions for collecting dietary data have been detailed previously [23–25]. The patients kept a self-completed dietary diary for a 3-day period, which included a dialysis day, a weekend day, and a nondialysis day. A licensed dietitian assisted the patients. Dietary data were obtained in person or through telephone interviews, and a 24-hour recall with standard household measuring tools was employed to confirm the data. Dietary energy and protein intake were calculated and normalized for body weight. Nutrient analysis software (Nutritionist Edition, Enhancement Plus 3, Version 2009) containing a Taiwanese food composition table as the nutrient database (Taichung, Taiwan) was used. Dietary protein intake was also estimated using the normalized protein nitrogen appearance (nPNA), as recommended by the National Kidney Foundation [26].

### Physical activity

During the dietetic interview, a self-description questionnaire designed by Liou et al. [27] was employed to measure subjects’ habitual physical activity. The levels of physical activity (light, moderate, intense, and very intense) and sleep were indicated as metabolic equivalents (METs), reported in kcal/day.

### Statistical analyses

All statistical analyses were performed using SAS software (Version 9.3). Before the hypothesis was tested, the Shapiro–Wilk test was employed to assess normality. Data were presented as the mean ± standard deviation, number with percentage, median with quartile range (Q1–Q3), correlation coefficient (r), and regression coefficient (β) with standard errors and 95% confidence intervals. According to our research, precisely what level of the CRP–Alb ratio was reciprocally associated with the risk of muscle wasting was unclear. Therefore, we empirically separated our patients by applying the 75th percentile of the CRP–Alb ratio as cutoff points and determined the differences between subjects according to the Student’s *t* test or Wilcoxon rank sum test. Correlations of the CRP–Alb ratio and other variables were assessed using Spearman’s rank correlation coefficients. *P* values < 0.05 were considered statistically significant. Univariate analysis through simple linear regression was performed to determine the predictors of muscle mass; SMM/wt. *P* values > 0.2 were reported as not significant. Stepwise multivariate analysis was followed to obtain the candidate final regression model. All univariate significant and nonsignificant relevant covariates and their interaction terms were selected. The significance level to entry (SLE) and significance level to stay (SLS) were set at 0.1. We also examined the variance inflation factor of each variable to assure the quality of the regression model.

## Results

### Baseline characteristics

The data for 91 adult HD patients (43 men and 48 women) are shown in Table 1. The mean age and dialysis vintage were 54.9 ± 6.6 years (range: 27–64 years) and 6.0 ± 5.8 years (range: 0.5–32.3 years), respectively. The proportions of comorbidities were as follows: diabetes in 42 patients (46.2%), hypertension in 30 patients (33.0%), and history of cardiovascular disease in 34 patients (37.4%). The serum albumin and CRP levels were 4.1 ± 0.3 mg/dL and 0.7 ± 1.2 mg/dL (median: 0.28; quartile range: 0.12–0.67), respectively. Only 29.7% of the patients had CRP > 0.5 mg/dL. Furthermore, the CRP–Alb ratio was 0.16 ± 0.29 (median: 0.07; quartile range: 0.025–0.162).

**Table 1:** Baseline demographics, anthropometric, laboratory, and dietary features of the study population (n = 91)^1^.

Items	Values
**Demographics**	
Male/female	43/48
Age, years	54.9 ± 6.6
Dialysis vintage, year	6.0 ± 5.8
DM, n (%)	42 (46.2)
Hypertension, n (%)	30 (33.0)
History of CVD, n (%)	34 (37.4)
Interdialytic weight gain, %	4.2 ± 1.2
**Anthropometry**	
Height, cm	161.5 ± 8.7
Body weight, kg	62.5 ± 12.9
BMI, kg/m^2^	23.9 ± 4.2
SMM, kg	24.3 ± 5.5
SMM/wt, %	39.1 ± 5.4
**Laboratory**	
Alb, g/dL	4.1 ± 0.3
Random blood glucose, mg/dL	121.6 ± 54.5
Insulin, μU/mL	18.1 ± 18.0
Blood urea nitrogen, mg/dL	77.5 ± 17.0
Creatinine, mg/dL	11.4 ± 2.0
TC, mg/dL	175.2 ± 37.9
TG, mg/dL	160.0 ± 109.8
High sensitivity CRP, mg/dL	0.7 ± 1.2
Intact parathyroid hormone, pg/L	366.7 ± 379.4
**Dietary intake**
Energy, kcal/day	1694.1 ± 499.3
Energy, kcal/kg/day	28.0 ± 9.9
Protein, g/day	61.7 ± 23.2
Protein, g/kg/day	0.8 ± 0.3
**Others**	
CRP–Alb ratio	0.16 ± 0.29
nPNA	1.4 ± 0.3
HOMA-IR	5.9 ± 8.0
GNRI	102.0 ± 4.8
eKt/V	1.6 ± 0.3
MET, kcal/day	599.8 ± 265.5

Abbreviations: DM, diabetes mellitus; CVD, cardiovascular disease; BMI, body mass index; SMM, skeletal muscle mass; SMM/wt, skeletal muscle mass normalized for body weight; Alb, albumin; TC, total cholesterol; TG, triglyceride; CRP, C-reactive protein; CRP–Alb ratio, ratio of C-reactive protein to albumin; nPNA, normalized protein nitrogen appearance; HOMA-IR, homoeostasis model assessment-estimated insulin resistance; GNRI, geriatric nutritional risk index; eKt/V, equilibrated Kt/V; MET, metabolic equivalent.

^1^Values are shown as the mean ± standard deviation or percentage.

### Comparison of muscle mass data based on quartile distribution of the CRP–Alb ratio

Table 2 displays the mean ages and parameters related to muscle mass according to the quartile distribution of the CRP–Alb ratio for the 91 consecutive adult patients. In this study, the 75th percentile of the CRP–Alb ratio corresponded to ≥ 0.22 and ≥ 0.11 for men and women, respectively. Regardless of sex, patients with higher CRP–Alb ratio levels tended to have lower muscle mass, indicated as SMM/wt, than did those with lower CRP–Alb ratio levels. However, the difference was nonsignificant (38.1% ± 6.9% vs. 39.4% ± 4.8%, *P* = 0.307), except with respect to creatinine (10.7 ± 2.2 mg/dL vs. 11.6 ± 2.0 mg/dL, *P* = 0.004). The results also indicated that the women in the 75th or higher percentiles of the CRP–Alb ratio had significantly lower SMM/wt levels and creatinine compared with the other women (SMM/wt: 33.2% ± 3.6% vs. 37.8% ± 5.0%, *P* = 0.002; creatinine: 9.7 ± 0.9 mg/dL vs. 10.9 ± 1.7 mg/dL, *P* = 0.005).

**Table 2:** Comparison of data stratified by CRP–Alb ratio in 91 consecutive adult patients with HD^1^^-^^3^.

	CRP–Alb < 75^th^ quartile			CRP–Alb ≥ 75^th^ quartile		
	All	Male	Female	All	Male	Female
n	68	32	36	23	11	12
Age, years	55.2 ± 6.7	55.8 ± 5.9	54.7 ± 7.3	54.0 ± 6.5	53.4 ± 8.1	54.7 ± 4.8
CRP–Alb ratio	0.05 ± 0.05	0.07 ± 0.06	0.04 ± 0.03	0.47 ± 0.46*	0.59 ± 0.59^$^	0.36 ± 0.29^#^
Creatinine, mg/dL	11.6 ± 2.0	12.4 ± 1.9	10.9 ± 1.7	10.7 ± 2.2*	11.8 ± 2.6	9.7 ± 0.9^#^
SMM/wt, %	39.4 ± 4.8	41.3 ± 3.8	37.8 ± 5.0	38.1 ± 6.9	43.4 ± 5.6	33.2 ± 3.6^#^

Abbreviations: SMM/wt, skeletal muscle mass normalized for body weight; HD, hemodialysis; CRP–Alb ratio, ratio of C-reactive protein and albumin.

^1^Values are shown as mean ± standard deviations.

^2^ The 75th percentile value of the CRP–Alb ratio was 0.22 and 0.11 for men and women, respectively.

^3^Statistical analyses were conducted using the Student’s *t* test or Wilcoxon rank sum test according to the distribution of data.

*Values were significantly different among all subjects with a CRP–Alb lower than the 75th percentile value (*P* < 0.05).

^$^Values were significantly different among male subjects with a CRP–Alb lower than the 75th percentile value (*P* < 0.05).

^#^Values were significantly different among female subjects with a CRP–Alb lower than the 75th percentile value (*P* < 0.05).

Notably, the CRP–Alb ratio was negatively correlated with SMM/wt (r = −0.33, *P* = 0.002), creatinine (r = −0.20, *P* = 0.056), eKt/V (r = −0.23, *P* = 0.033), and total energy intake (r = −0.22, *P* = 0.046) after adjustment for sex and age. In addition, the CRP–Alb ratio was positively correlated with body weight (r = 0.32, *P* = 0.003), BMI (r = 0.28, *P* = 0.008), random blood glucose (r = 0.27, *P* = 0.012), insulin (r = 0.22, *P* = 0.043), and HOMA-IR (r = 0.27, *P* = 0.011).

### Determining the confounders related to muscle mass

Univariate analysis was used to determine the factors associated with muscle mass in patients undergoing HD (Table 3). We determined that the CRP–Alb ratio showed a significant independent association with SMM/wt (β = −2.609, *P* < 0.2). After further adjusting for sex and age, the CRP–Alb ratio was still inversely and significantly associated with muscle mass (β = −3.91, *P* = 0.022). Other general factors, namely energy intake (β = 0.16, *P* = 0.002), BMI (β = −0.76, *P* < 0.0001), and HOMA-IR (β = −0.18, *P* = 0.005), were significant predictors of muscle mass.

**Table 3:** Simple regression analysis using SMM/wt as a dependent variable^1^.

	β	SE	95% CI		*P*
			Minimum	Maximum	
**Anthropometry**					
Height	0.299	0.058	0.183	0.414	< .0001
Body weight	−0.079	0.044	−0.165	0.008	0.074
BMI	−0.694	0.116	−0.924	−0.465	< .0001
**Laboratory**					
Creatinine	0.751	0.267	0.220	1.282	0.006
Albumin	3.284	1.936	−0.564	7.132	0.093
CRP	−0.574	0.462	−1.492	0.344	0.217
**Dietary**					
Energy	0.003	0.001	0.001	0.005	0.009
Protein	0.035	0.024	−0.014	0.083	0.156
**Others**					
eKt/V	−3.321	1.792	−6.882	0.240	0.067
HOMA-IR	−0.155	0.069	−0.293	−0.017	0.028
CRP–Alb ratio	−2.609	1.926	−6.436	1.217	0.179

Abbreviations: SMM/wt, skeletal muscle mass normalized for body weight; BMI, body mass index; CRP, C-reactive protein; CRP–Alb ratio, ratio of C-reactive protein and albumin; eKt/V, equilibrated Kt/V; HOMA-IR, homeostatic model assessment-insulin resistance.

^1^Values are shown as regression coefficients (β) with standard error and a 95% confidence interval. *P* value > 0.2 is reported as not significant in the univariate regression analysis.

We analyzed the independent contributing factors for SMM/wt through stepwise multiple regression analysis. Variables retained in the model are presented in Table 4. The CRP–Alb ratio and HOMA-IR were the independent risk determinants for SMM/wt in the adult patients undergoing HD (adjusted R^2^ = 0.32). Moreover, this candidate final regression model was not affected by the multicollinearity because all the variance inflation factors were less than 2.

**Table 4:** Stepwise multiple regression analysis using SMM/wt as a dependent variable^1^.

Items	β	95% CI		*P*	Adjusted R^2^
		Minimum	Maximum		
Constant	38.90	29.97	47.88	< .0001	0.32
CRP–Alb ratio	−3.84	−7.05	−0.64	0.019	
HOMA-IR	−0.18	−0.30	−0.06	0.004	

Abbreviations: SMM/wt, skeletal muscle mass normalized for body weight; CRP–Alb ratio, ratio of C-reactive protein and albumin; HOMA-IR, homeostatic model assessment-insulin resistance.

^1^Values are shown as regression coefficients (β) with a 95% confidence interval and adjusted R square (R^2^). The SLE and SLS of any potential factor or interaction in the stepwise variable selection were set at 0.1. Variables such as sex, age, and total energy intake were forced as a regressor during stepwise selection. Variables retained in the model are presented in the table.

## Discussion

The results of this cross-sectional study reveal that an elevated CRP–Alb ratio is significantly associated with decreased muscle mass, which was indicated as SMM/wt (Table 3). This relationship was maintained in the multivariable regression after adjustment for univariate significant and nonsignificant relevant covariates for muscle mass loss, namely age, sex, comorbidities such as HOMA-IR, physical activity, and dietary energy intake (Table 4). These results suggest that monitoring serum CRP and albumin levels may be an inexpensive, alternative, and useful method for evaluating the risk profiles for muscle wasting of ESRD patients. However, our findings may not be may not be generalizable to the entire HD population. According to our research, similar findings have not previously been published. Further large-scale studies are necessary to assess whether our observations can be extrapolated to address this issue.

Using the CRP–Alb ratio as an independent risk determinant for muscle mass in adults with ESRD has several clinical implications. Huang et al. determined that patients undergoing maintenance HD with a fluctuating CRP–Alb ratio tend to have longer subsequent hospital stays and higher mortality risk [10]. Muscle wasting is an ineffective prognostic factor in dialysis patients [1, 28]. In a contemporary cohort of 117,683 adult HD patients [29], inverse relationships between fat-free muscle mass and mortality were identified. We hypothesize that using the CRP–Alb ratio as an alternative surrogate marker of muscle mass in ESRD patients provides not only indications of their inflammatory and nutritional statuses, but also of comorbidity risk, such as protein energy wasting or malnutrition-inflammation complex syndrome.

Precisely what level of the CRP–Alb ratio is associated with the risk of muscle wasting is unclear. Fairclough et al. proposed the concept of the CRP–Alb ratio for acute medical admissions [15]. Kim et al. asserted that a CRP–Alb ratio > 5.09 yielded the highest sensitivity and specificity in predicting the 180-day mortality of patients with sepsis [30]. Ranzani et al. observed lower survival in septic patients with a CRP–Alb ratio > 2 [19]. According to our research, no previous studies have examined the CRP–Alb ratio as a predictive index of muscle mass, the candidate prognostic factor in patients with ESRD. We empirically stratified our patients according to the 75th percentile of the CRP–Alb ratios, determining that the subjects with decreased CRP–Alb ratios were associated with higher muscle mass, which was indicated as SMM/wt and creatinine (Table 2). Future longitudinal studies should elucidate the diagnostic performance of the CRP–Alb ratio in determining the muscle mass of ESRD patients.

Another notable result is that the CRP–Alb ratios increased with decreasing muscle mass in the female participants, but not in the male participants. The CRP–Alb ratio was also associated with body weight, BMI, random blood glucose, insulin, and HOMA-IR. In general, women have more fat mass and lower muscle mass than men do. Kim et al. revealed that women have a higher prevalence of sarcopenic obesity than men do [12]. Schrager et al. asserted that sarcopenic obesity is significantly associated with elevated levels of CRP [31]. Furthermore, the accumulation of adipose tissue is associated with the number of adipocytokines that stimulate the liver to synthesize acute phase proteins and to increase the serum CRP level [8]. In our study, sex and age were applied as covariates for Spearman’s rank correlation and stepwise selection. Prospective studies monitoring the change in the CRP–Alb ratio over time regarding sex difference are merited.

The link between IR and muscle mass that was determined in the present study could indicate that insulin plays a role in regulating body protein mass [32]. Consistent with previous studies, Thage found a high prevalence of uremic sarcopenia in patients with diabetes undergoing dialysis [33]. Pupim et al. determined that the presence of diabetes is the crucial determinant for muscle mass loss, independent of other confounders such as age, sex, and status of inflammation [34]. In a 4.6-year follow-up cohort study [35], Lopez Teros et al. observed that hyperinsulinemia was an early predictor of IR, associated with the loss of appendicular SMM in 147 well-functioning older subjects. The possible mechanisms underlying muscle mass and IR were (1) muscle tissue as the primary site for IR [36]; (2) the IR-initiated reduction of the activity of the Class I phosphatidylinositol 3-kinase–Akt pathway, which triggers the activation of the proteasome-ubiquitin pathway [37]; and (3) the activation of the apoptosis regulator Bax, resulting in the stimulation of caspase-3 activity [38]. Therefore, the treatment of this metabolic abnormality could profoundly influence muscle wasting.

Several limitations are worth noting. First, this was a cross-sectional study with a relatively small sample size, and our results describe only the association and not the causality between the CRP–Alb ratio and muscle mass in adult patients undergoing HD. The adult patients in this study were relatively healthy: only 2.2% (n = 2) and 4.4% (n = 4) of them had albumin levels < 3.5 g/dL and GNRI values < 91.2, respectively, suggesting lower prevalence and less severe levels of muscle wasting. The results are more likely to be observed in the context of our study population demographics, which may limit the generalizability of the results. Additional validation studies are necessary to assess whether our observations can be extrapolated to general HD patients of advanced age, different races, alternative modalities of renal replacement therapy, and different statuses of inflammation and muscle wasting. However, we calculated the *Post-hoc* statistical power for the multiple regression on the basis of the following parameters: the observed probability level (value = 0.1: based on the SLS and SLS), the number of predictors (n = 2: CRP–Alb ratio and HOMA-IR; Table 4), the observed R^2^ (value = 0.32; Table 4), and the sample size (n = 91). The observed statistical power of our final multiple linear regression supports the distribution of the CRP–Alb ratio and muscle mass in this study. Second, we estimated muscle mass through a BIA alternative to dual-energy X-ray absorptiometry, computed tomography, and magnetic resonance imaging (MRI). Because the accuracy of BIA to measure body compartments is diminished in edema states, particularly with fluctuating volume and electrolyte status, the measurement procedures for assessing body composition were conducted after HD sessions. Moreover, patients in this study regularly underwent a thrice weekly HD regimen with an adequate dialysis dose, indicated as an equilibrated Kt/V (eKt/V) of at least 1.2. Kaysen et al. also revealed that the results obtained through BIA estimation of total body and limb muscle mass correlated with those obtained through MRI [39].

In conclusion, the present study indicated that the CRP–Alb ratio, applied as a routine clinical marker of inflammation and nutritional status, is independently associated with muscle mass in adult patients undergoing HD. We demonstrated the simplicity, economy, convenience, and objectiveness of the CRP–Alb ratio, and that this ratio is an independent risk determinant for muscle wasting. Our results may be valuable for clinicians who manage nutritional assessment, and may enable the development of targeted strategies for treating ESRD patients.

## Supporting Information

S1 File The certification of Taipei Medical University Joint Institutional Review Board. (PDF)
