# Supplementary material for: Ratio of C-Reactive Protein to Albumin Predicts Muscle Mass in Adult Patients Undergoing Hemodialysis
Source: PLoS One. 2016 Oct 21;11(10):e0165403. doi: 10.1371/journal.pone.0165403 (PMC5074567; doi:10.1371/journal.pone.0165403)
Supplement: S1 File — (PDF) [file pone.0165403.s001.pdf]

臺北醫學大學暨附屬醫院聯合人體研究倫理委員會

TMU-Joint Institutional Review Board

通過證明函 － 執行效期展延

開立日期：民國 104 年 2 月 18 日

本會編號：201302024
計畫名稱：改善血液透析患者完全營養照護品質對心血管疾病預後之相關性研究
計畫主持人：楊淑惠
協同主持人：陳錫賢、蘇千田、陳作孝、許永和
試驗/研究機構：臺北醫學大學附設醫院、臺北市立萬芳醫院、衛生福利部雙和醫院、
溫昕護理之家、溫昕老人長期照顧中心（養護型）、景美醫院附設護理之家
計畫書版本/日期：Version 15.0 / 07/02/2014
受試(訪)者同意書版本/日期： Version 7.0 / 06/13/2014
個案報告表版本/日期：Version 3 / 2013/3/15

上述計畫之期中報告，業經第 104-03-3 次會期審查，特此證明。有效期限自民國 104 年 3 月 20 日至民國 105 年 3 月 19 日。試驗/研究期間應接受本會之監督。

依據衛生福利部與相關規定， 後續追蹤程序及要求如下：

1. 期中報告：本計畫期中繳交頻率為**每 12 個月**，應於有效期限到期**前二個月（民國 105 年 1 月 19 日）**繳交期中報告。有效期限屆滿時若尚未通過期中報告與效期展延審查者，試驗/研究不得繼續執行。
2. 追蹤報告：應依本會審查通過時之決議，定期繳交至本會審查。
3. 結案報告：試驗/研究完成後，應將執行情形及結果依結案報告表要求送至本會審查。**試驗/研究結束後三個月仍未繳交者，本會得撤銷本通過證明函，亦即撤銷本試驗/研究之核准，亦將依本會作業程序暫停主持人(含任何參與形式)申請新試驗/研究案之審查三個月。**
4. 嚴重不良事件(SAE)報告：執行人體試驗或臨床試驗之主持人應根據衛生署「藥品優良臨床試驗準則」和「嚴重藥物不良反應通報辦法」規定，辦理相關事宜。

林時宜主任委員

Certificate of TMU-JIRB Approval

TMU-JIRB No.：201302024
Protocol Title：The association between improved nutritional care and the prognosis of cardiovascular disease for hemodialysis patients
Principal Investigator：Yang, Shwu-Huey
Investigator：Chen Hsi-Hsien, CHIEN-TIEN SU, Tso-Hsiao Chen, Yung-Ho Hsu
Study site：Taipei Medical University Hospital, Wanfang Hospital, TMU-Shuang-Ho Hospital, Win-Life Nursing Home, Win-Life Long-term Care Center for elderly, Jing-Mei Hospital, nursing home
Protocol Version/Date：Version 15.0 / 07/02/2014
Informed Consent Forms：Version 7.0 / 06/13/2014
Case Report Forms：Version 3 / 2013/3/15

The above study's progress report has been approved by TMU-Joint Institutional Review Board, valid till March 19th 2016, and must be monitored by TMU-JIRB.

Shyr-Yi Lin , M.D., Ph.D
Chairman

臺北醫學大學暨附屬醫院
聯合人體研究倫理委員會
Taipei Medical University
Joint Institutional Review Board

本會組織與執行皆符合適用法規
The TMU-Joint Institutional Review Board performs its functions according to written operating procedures and complies with GCP and with the applicable regulatory requirements.

臺北醫學大學暨附屬醫院聯合人體研究倫理委員會
TMU-Joint Institutional Review Board

通過證明函－修正案(簡易審查)

開立日期：民國 **103** 年 **7** 月 **27** 日

本會編號：201302024
計畫名稱：改善血液透析患者完全營養照護品質對心血管疾病預後之相關性研究
計畫主持人：楊淑惠
協同主持人：陳錫賢、蘇千田、陳作孝、許永和
試驗/研究機構：臺北醫學大學附設醫院、臺北市立萬芳醫院、衛生福利部雙和醫院、溫昕護理之家、溫昕老人長期照顧中心（養護型）、景美醫院附設護理之家
申請書版本/日期：07/11/2014
計畫書版本/日期：Version 15.0 / 07/02/2014
計畫書摘要版本/日期：Version 12.0 / 07/02/2014
受試者同意書版本/日期：Version 7.0 / 06/13/2014

上述計畫之修正，業經第 103-07-3 次會期審查，特此證明。有效期限自**民國 103 年 7 月 27 日至民國 104 年 3 月 19 日**。試驗/研究期間應接受本會之監督。

依據衛生福利部與相關規定，後續追蹤程序及要求如下：

1. 期中報告：本計畫期中繳交頻率為每 12 個月，應於有效期限到期前二個月（**民國 104 年 1 月 19 日**）繳交期中報告。有效期限屆滿時若尚未通過期中報告與效期展延審查者，試驗/研究不得繼續執行。
2. 追蹤報告：應依本會審查通過時之決議，定期繳交至本會審查。
3. 結案報告：試驗/研究完成後，應將執行情形及結果依結案報告表要求送至本會審查。試驗/研究結束後三個月仍未繳交者，**本會得撤銷本通過證明函，亦即撤銷本試驗/研究之核准，亦將依本會作業程序暫停主持人(含任何參與形式)申請新試驗/研究案之審查三個月**。
4. 嚴重不良事件(SAE)報告：執行人體試驗或臨床試驗之主持人應根據衛生署「藥品優良臨床試驗準則」和「嚴重藥物不良反應通報辦法」規定，辦理相關事宜。

主任委員
薛瑞元 薛瑞元

臺北醫學大學暨附屬醫院
聯合人體研究倫理委員會
Taipei Medical University
Joint Institutional Review Board

臺北醫學大學暨附屬醫院聯合人體研究倫理委員會
TMU-Joint Institutional Review Board

通過證明函 － 執行效期展延

開立日期：民國 103 年 1 月 20 日

本會編號：201302024
計畫名稱：改善血液透析患者完全營養照護品質對心血管疾病預後之相關性研究
總計畫主持人/計畫主持人：楊淑惠
協同主持人：陳錫賢、蘇千田
試驗/研究機構：臺北醫學大學附設醫院
計畫書版本/日期：Version 12 / 2013/2/27
受試(訪、檢)者同意書版本/日期：Version 4 / 2013/3/3
個案報告表版本/日期：Version 3 / 2012/3/15

上述計畫之期中報告已通過本會審查，將於第 103-02-2 次會期追認，特此證明。有效期限自**民國 103 年 1 月 20 日至民國 104 年 3 月 19 日**，為期一年。試驗/研究期間應接受本會之監督。

依據衛生署與 ICH-GCP 規定，後續追蹤程序及要求如下：
1. 期中報告：本計畫期中繳交頻率為**每 12 個月**，應於有效期限到期**前二個月（民國 104 年 1 月 19 日）**繳交期中報告。有效期限屆滿時若尚未通過期中報告與效期展延審查者，試驗/研究不得繼續執行。
2. 追蹤報告：應依本會審查通過時之決議，定期繳交至本會審查。
3. 結案報告：試驗/研究完成後，應將執行情形及結果依結案報告表要求送至本會審查。試驗/研究**結束後三個月**仍未繳交者，**本會得撤銷本通過證明函，亦即撤銷本試驗/研究之核准，亦將依本會作業程序暫停主持人(含任何參與形式)申請新試驗/研究案之審查三個月**。
4. 嚴重不良事件(SAE)報告：執行人體試驗或臨床試驗之主持人應根據衛生署「藥品優良臨床試驗準則」和「嚴重藥物不良反應通報辦法」規定，辦理相關事宜。

主任委員
薛瑞元 (signature)

Certificate of TMU-JIRB Approval

TMU-JIRB No.：201302024
Protocol Title：The association between improved nutritional care and the prognosis of cardiovascular disease for hemodialysis patients
Principle Investigator：Yang, Shwu –Huey
Investigators：Chen His-Hsien, Chien-Tien Su
Study site：Taipei Medical University
Protocol Version/Date：Version 12 / 2013/2/27
Informed Consent Forms：Version 4 / 2013/3/3
Case Report Forms：Version 3 / 2012/3/15

The above study's mid-term report has been approved by TMU-Joint Institutional Review Board, validity is from January 20th, 2014, till March 19th, 2015, and must be monitored by TMU-JIRB.

Jui-Yuan, Hsueh , M.D.,
Chairman (signature)

臺北醫學大學暨附屬醫院
聯合人體研究倫理委員會
Taipei Medical University
Joint Institutional Review Board

臺北醫學大學暨附屬醫院聯合人體研究倫理委員會
TMU-Joint Institutional Review Board

通過證明函－修正案(簡易審查)

開立日期：民國 102 年 12 月 19 日

本會編號：201302024
計畫名稱：改善血液透析患者完全營養照護品質對心血管疾病預後之相關性研究
計畫主持人：楊淑惠
協同主持人：陳錫賢、蘇千田、陳作孝
試驗/研究機構：臺北醫學大學附設醫院、臺北市立萬芳醫院
申請書版本/日期：11/25/2013
計畫書版本/日期：Version 14.0 / 12/13/2013
計畫書摘要版本/日期：Version 11.0 / 12/13/2013
受試者同意書版本/日期：Version 6.0 / 12/13/2013

上述計畫之修正已通過本會審查，將於第 102-12-3 次會期追認，特此證明。有效期限自民國 102 年 12 月 19 日至民國 103 年 3 月 19 日。試驗/研究期間應接受本會之監督。

依據衛生署與 ICH-GCP 規定，後續追蹤程序及要求如下：

1. 期中報告：本計畫期中繳交頻率為**每 12 個月**，應於有效期限到期**前二個月**（**民國 103 年 1 月 19 日**）繳交期中報告。有效期限屆滿時若尚未通過期中報告與效期展延審查者，試驗/研究不得繼續執行。
2. 追蹤報告：應依本會審查通過時之決議，定期繳交至本會審查。
3. 結案報告：試驗/研究完成後，應將執行情形及結果依結案報告表要求送至本會審查。試驗/研究**結束後三個月**仍未繳交者，**本會得撤銷本通過證明函，亦即撤銷本試驗/研究之核准，亦將依本會作業程序暫停主持人(含任何參與形式)申請新試驗/研究案之審查三個月**。
4. 嚴重不良事件(SAE)報告：執行人體試驗或臨床試驗之主持人應根據衛生署「藥品優良臨床試驗準則」和「嚴重藥物不良反應通報辦法」規定，辦理相關事宜。

主任委員
薛瑞元 [signature]

Certificate of TMU-JIRB Approval

TMU-JIRB No.：201302024
Protocol Title：The association between improved nutritional care and the prognosis of cardiovascular disease for hemodialysis patients
Principal Investigator：Yang, Shwu-Huey
Investigator：Chen Hsi-Hsien, CHIEN-TIEN SU, Tso-Hsiao Chen
Study site：Taipei Medical University Hospital, Wanfang Hospital
Application Form Version/Date：11/25/2013
Protocol Version/Date：Version 14.0 / 12/13/2013
Summary Version/Date：Version 11.0 / 12/13/2013
Informed Consent Forms：Version 6.0 / 12/13/2013

The amendment of above study has been approved by the TMU-Joint Institutional Review Board, valid till March 19th 2014, and must be monitored by TMU-JIRB.

Jui-Yuan, Hsueh , M.D.,
Chairman
[signature]

臺北醫學大學暨附屬醫院聯合人體研究倫理委員會
Taipei Medical University Joint Institutional Review Board

本會組織與執行皆符合 ICH-GCP 及適用法規
The TMU-Joint Institutional Review Board performs its functions according to written operating procedures and complies with GCP and with the applicable regulatory requirements.

臺北醫學大學暨附屬醫院聯合人體研究倫理委員會
TMU-Joint Institutional Review Board

通過證明函－簡易審查案

開立日期：民國 102 年 3 月 20 日

本會編號：201302024
計畫名稱：改善血液透析患者完全營養照護品質對心血管疾病預後之相關性研究
總計畫主持人/計畫主持人：楊淑惠
計畫書版本/日期：Version 12 / 2013/2/27
受試(訪、檢)者同意書版本/日期：Version 4 / 2013/3/3
個案報告表版本/日期：Version 3 / 2012/3/15

上述計畫已通過本會簡易審查程序，將於第 102-04-3 次會期追認，特此證明。有效期限至民國 103 年 3 月 19 日，為期一年。試驗/研究期間應接受本會之監督。

依據衛生署與 ICH-GCP 規定，後續追蹤程序及要求如下：

1. 期中報告：本計畫期中繳交頻率為**每 12 個月**，應於有效期限到期前二個月（**民國 103 年 1 月 19 日**）繳交期中報告。有效期限屆滿時若尚未通過期中報告與效期展延審查者，試驗/研究不得繼續執行。
2. 追蹤報告：應依本會審查通過時之決議，定期繳交至本會審查。
3. 結案報告：試驗/研究完成後，應將執行情形及結果依結案報告表要求送至本會審查。試驗/研究結束後三個月仍未繳交者，**本會得撤銷本通過證明函，亦即撤銷本試驗/研究之核准，亦將依本會作業程序暫停主持人(含任何參與形式)申請新試驗/研究案之審查三個月**。
4. 嚴重不良事件(SAE)報告：執行人體試驗或臨床試驗之主持人應根據衛生署「藥品優良臨床試驗準則」和「嚴重藥物不良反應通報辦法」規定，辦理相關事宜。

主任委員
薛瑞元 薛瑞元

Certificate of TMU-JIRB Approval

TMU-JIRB No.：201302024
Protocol Title：The association between improved nutritional care and the prognosis of cardiovascular disease for hemodialysis patients
Principle Investigator：Yang, Shwu -Huey
Protocol Version/Date：Version 12 / 2013/2/27
Informed Consent Forms：Version 4 / 2013/3/3
Case Report Forms：Version 3 / 2012/3/15

The above study has been approved by expedited review process of the TMU-Joint Institutional Review Board, valid till January 19th, 2014, and must be monitored by TMU-JIRB.

Jui-Yuan, Hsueh , M.D.,
Chairman

臺北醫學大學暨附屬醫院
聯合人體研究倫理委員會
Taipei Medical University
Joint Institutional Review Board

本會組織與執行皆符合 ICH-GCP 及適用法規
The TMU-Joint Institutional Review Board performs its functions according to written operating procedures and complies with GCP and with the applicable regulatory requirements.
